# Ectopic Deciduous Maxillary Tooth in the Nasal Cavity Following Trauma

**DOI:** 10.7759/cureus.14154

**Published:** 2021-03-28

**Authors:** Vivek Dokania, Harish Kinnera, Shweta S, Neeraj Shetty, Ninad Gaikwad

**Affiliations:** 1 Department of Otolaryngology-Head & Neck Surgery, Hinduhridaysamrat Balasaheb Thackeray (HBT) Medical College and Dr. Rustom Narsi (RN) Cooper Municipal General Hospital, Mumbai, IND

**Keywords:** intranasal tooth, deciduous teeth, trauma

## Abstract

Several cases of the ectopic supernumerary tooth in the nasal cavity have been reported; however, an eruption of the primary maxillary tooth in the nose following trauma is extremely rare. Clinical evaluation and discriminating features on CT imaging, particularly with bone window setting, are sufficient to confirm the diagnosis. We discuss a case of deciduous central maxillary incisor in the nasal cavity and specifically focus on its clinical and radiological presentation. None of the previous authors have discussed probable theories of the post-traumatic intranasal tooth; we consider two possible theories which might be responsible for the ectopic intranasal tooth after trauma. Additionally, we discuss pertinent features which help distinguish ectopic deciduous tooth from the supernumerary tooth.

## Introduction

The first case of the ectopic intranasal tooth was described by Albins in 1754 [1]. Subsequently, many cases were reported in the 19th and early 20th centuries [2]. Most of these cases consisted of a supernumerary tooth. Very few reports of a post-traumatic deciduous tooth in the nasal cavity have been reported in the English literature. The ectopic eruption can result from trauma, sepsis, osteomyelitis, developmental abnormalities, over-crowding, and genetic factors. Yet, the etiology of the intranasal tooth is not well understood, and no etiological factor has been identified in a majority of cases. We assume two possible theories for ectopic nasal tooth following trauma: (1) a sudden displacement of a detached tooth in the nasal cavity induced by a forceful trauma, or (2) an upward inversion of maxillary tooth crown after a maxillo-facial trauma, and followed by a gradual caput growth with an eruption of the tooth from the floor of the nasal cavity. We present a case of the deciduous central maxillary tooth in the nasal cavity of a child, noticed few months after the initial trauma, and consider the “inversion theory,” as a possible reason.

## Case presentation

**Figure 1:**
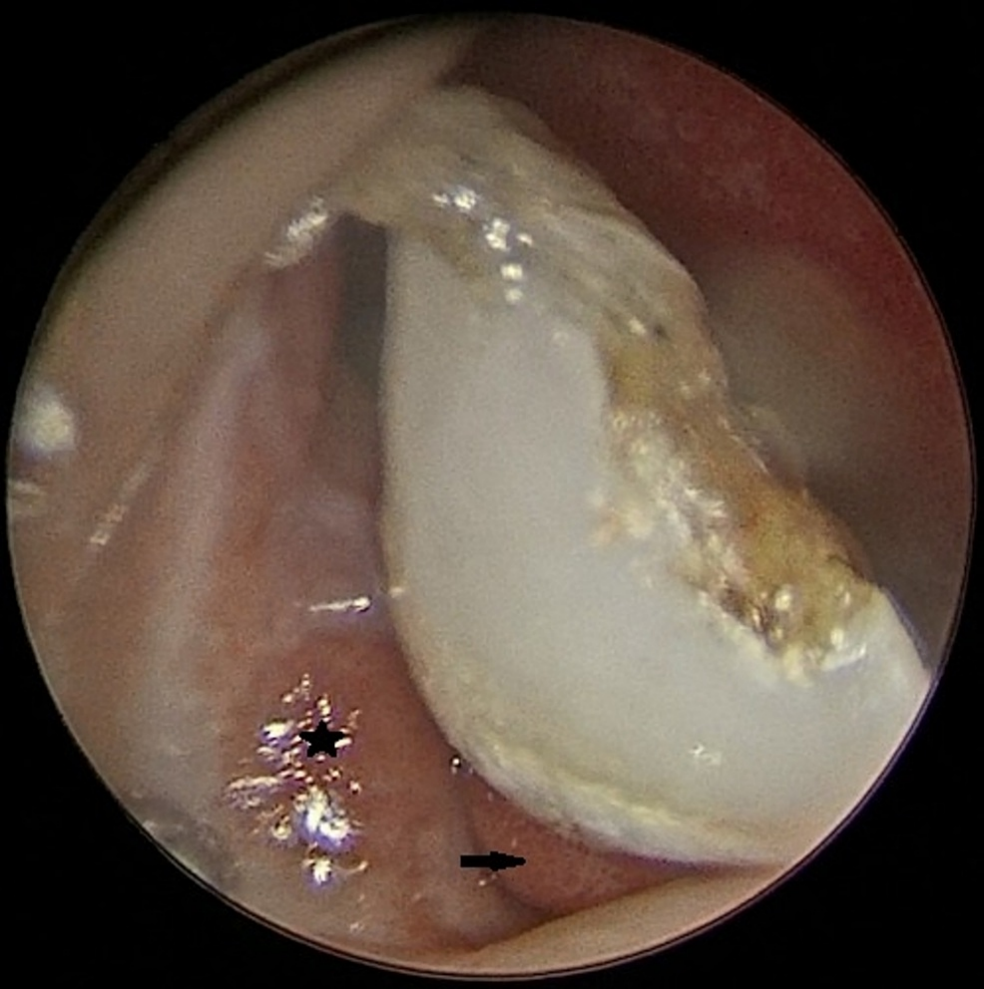
Endoscopic nasal examination shows a white mass arising from the floor of right nasal cavity. The mass is related laterally to inferior turbinate (black star) and is surrounded by granulation tissue at its base (black arrow).

A four-year-old boy presented with complaints of recurrent epistaxis and a bony white mass in the right nostril, in November 2020. The child’s mother initially noticed a small intranasal mass in March 2020, which she said has gradually increased to the current noticeable size. She did not visit a hospital earlier because of the coronavirus disease-19 (COVID-19) pandemic. However, the epistaxis episodes became frequent in the last few days. The child’s mother gave a history of loss of a maxillary central incisor following a dental trauma in November 2019, due to a fall. The mother was not able to find the tooth and was considered lost. A few weeks later, she felt a small and hard bump beneath the right nasolabial skin, which gradually kept drifting nasally, until she noticed a white mass in the right nasal cavity. On endoscopic nasal examination, a bony-hard, white mass was seen erupting from the floor of the right nostril and medial to the anterior edge of the inferior turbinate. A dome of granulation tissue was seen surrounding the base of the mass (Figure 1).

**Figure 2:**
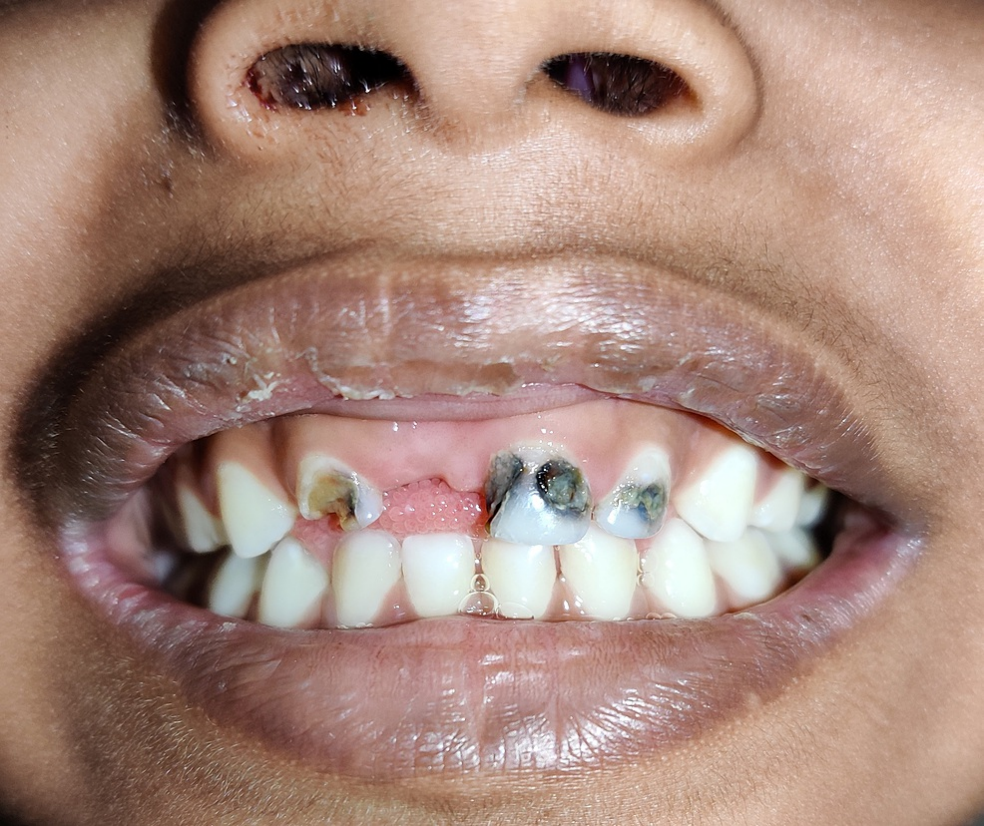
Oro-dental examination shows an absence of primary central maxillary incisor on the right side.

Oral examination showed a missing right upper central incisor tooth (Figure 2).

**Figure 3:**
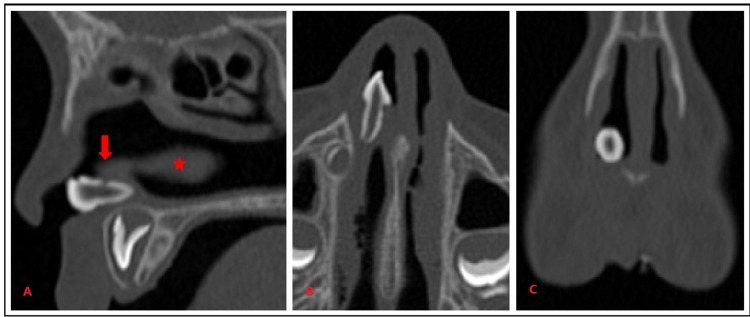
CT scan with bone window setting shows a homogeneous high attenuation lesion adjacent to inferior turbinate (red star), and surrounded by soft tissue at its base corresponding to the granulation tissue (red arrow) (A). The lesion showed a central slit-like radiolucency in axial view (B) and a focal central radiolucency in coronal view (C), both corresponding to pulp space.

The CT scan showed a radio-opaque mass located in the floor of the right nasal cavity between the inferior turbinate and the nasal septum. The mass was surrounded posteriorly by soft tissue, consistent with the clinical finding of granulation tissue. With the bone window setting of a 500-HU window length (WL) and 1500-HU window width (WW), the lesion was noted to have a homogeneous high attenuation equivalent to that of the teeth and its root was buried in the mucosa of the nasal floor but was not surrounded by any bony socket. A slit-like cavity in sagittal cut and a focal central radiolucency in coronal cut, both correlating to the pulp cavity was seen (Figure 3). These findings confirmed a diagnosis of the nasal tooth.

**Figure 4:**
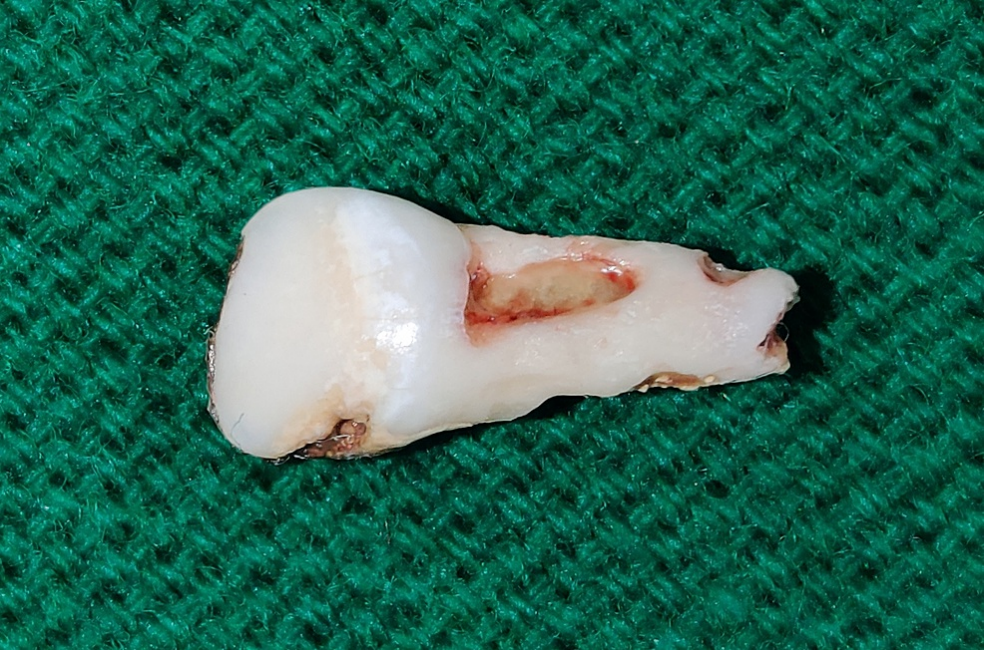
Excised intranasal tooth.

The patient underwent endoscopic removal of the nasal tooth. Gross examination of the excised tooth showed a root/crown ratio of ∼2, and the mesiodistal diameter of the crown exceeded its cervico-incisal length (Figure 4). Both these findings, along with an absence of upper central incisor on clinical examination, confirmed the nasal tooth which is consistent with a deciduous maxillary central incisor. 

## Discussion

Ectopic teeth may be present in various sites of the maxillofacial region including maxillary sinus, palate, mandibular condyle, coronoid process, orbit, nasal cavity, or through the skin [3]. In most of the cases, no etiological factor has been found, although, trauma, infection, developmental disturbances, and genetic factors have been proposed in a small number of cases [4]. Eruption of the tooth in the nasal cavity is a relatively rare phenomenon. Nonetheless, an eruption of a primary or permanent tooth in the nasal cavity following trauma is extremely rare, and most of the reported cases of the ectopic intranasal tooth either comprised of a supranumerary tooth or had a non-traumatic etiology [5,6].

Clinically, nasal teeth might be asymptomatic and detected incidentally, or they may present with a variety of signs, symptoms, or complications: nasal obstruction, nasal discharge, epistaxis, rhinitis caseosa, oro-nasal fistula, facial pain, mild fever, septal abscess, or external nasal deviation [3].

A clinical evaluation combined with a CT scan (preferably using a bone-specific reformat algorithm) is usually sufficient to diagnose an ectopic primary/permanent nasal tooth. Clinically, an intranasal tooth may be seen as a white mass and surrounded by granulation tissue and/or debris. CT scan may show a radio-opaque lesion with the same attenuation as that of the oral teeth (bone density corresponds to +1000 HU). With the bone window setting, the central pulp space may be seen as a radiolucent slit or spot, depending on the inclination of the teeth [7]. Nevertheless, a supranumerary nasal tooth, unlike a primary/permanent tooth, is deformed in appearance and usually has a peg-,cone-, or triangle-shaped crown, and mostly lacks a central pulp space [8]. These features of a supranumerary nasal tooth make it tough to diagnose clinically or on CT imaging.

Removal of nasal teeth is generally recommended to relieve the symptoms and prevent complications. An endoscopic-endonasal, a conventional-endonasal, or a transoral approach is advocated, depending on the age of the patient, depth of eruption, presence/absence of any bony socket, and surgeon’s experience in the endoscopic nasal surgery [3,7-10].

## Conclusions

Deciduous maxillary tooth in the nasal cavity following trauma is extremely rare. Characteristic clinical features and discriminating imaging findings on CT scan are sufficient to diagnose an ectopic intranasal primary tooth. Gradual nasal growth after trauma-induced inversion of the oral denture (inversion theory) is a possibility in a patient with a remote history of trauma, and loss of denture which was later not found after trauma. Gross examination of the excised mass further confirms the type of deciduous tooth and distinguishes it from a supernumerary tooth.

## References

[1] Kuroda H, Tsutsumi K, Tomisawa H, Koizuka I (2003). A case of an inverted tooth in the nasal cavity. Auris Nasus Larynx.

[2] Accorona R, Colombo G, Ferrari M, Fazio E, Bolzoni-Villaret A (2020). Inverted supernumerary intranasal teeth as unusual indications of endoscopic surgery. Iran J Otorhinolaryngol.

[3] Kim DH, Kim JM, Chae SW, Hwang SJ, Lee SH, Lee HM (2003). Endoscopic removal of an intranasal ectopic tooth. Int J Pediatr Otorhinolaryngol.

[4] van Essen TA, van Rijswijk JB (2013). 'Intranasal toothache': case report. J Laryngol Otol.

[5] Gilbride MJ, Smith WP (2005). Eruption of teeth in the nose following trauma to the primary and permanent dentitions. Br Dent J.

[6] Johnson AP (1981). A case of an intranasal canine tooth. J Laryngol Otol.

[7] Chen A, Huang JK, Cheng SJ, Sheu CY (2002). Nasal teeth: report of three cases. AJNR Am J Neuroradiol.

[8] Lee FP (2001). Endoscopic extraction of an intranasal tooth: a review of 13 cases. Laryngoscope.

[9] Gupta YK, Shah N (2001). Intranasal tooth as a complication of cleft lip and alveolus in a four year old child: case report and literature review. Int J Paediatr Dent.

[10] Sammartino G, Trosino O, Perillo L, Cioffi A, Marenzi G, Mortellaro C (2011). Alternative transoral approach for intranasal tooth extraction. J Craniofac Surg.

